# Fresh Properties of EVA-Modified Cementitious Mixtures for Use in Additive Construction by Extrusion

**DOI:** 10.3390/ma12142292

**Published:** 2019-07-18

**Authors:** Kyu-Seok Yeon, Kwan Kyu Kim, Jaeheum Yeon, Hee Jun Lee

**Affiliations:** 1 Department of Regional Infrastructure Engineering, Kangwon National University, Chuncheon, Gangwon 24341, Korea; 2 North Gyeonggi Branch, Joongbu Division, Korea Conformity Laboratories, Pocheon, Gyeonggi 11184, Korea; 3 Department of Engineering and Technology, Texas A&M University-Commerce, Commerce, TX 75429, USA

**Keywords:** additive construction by extrusion, EVA-modified cementitious mixtures, fresh properties, flowability, extrudability, buildability, open time

## Abstract

In this study, the fresh properties of ethylene–vinyl acetate (EVA)-modified cementitious mixtures were experimentally investigated to evaluate the feasibility of this type of material being used in additive construction by extrusion (ACE). The EVA/cement ratio was a main variable to determine the properties, including flowability, extrudability, buildability, and open time. According to the flow test results, the optimized flow of the EVA-modified cementitious mixtures was found to be 65% for buildability. This excellent flowability could be achieved because the flow increased as the EVA/cement ratio increased; conversely, the extrudability was slightly reduced when the EVA/cement ratio increased. However, if the flow of the EVA-modified cementitious mixtures was maintained at 65%, ACE could be achieved without significant issues. In addition, the height of the additive concrete walls created was not substantially reduced after printing of these mixtures, even though different EVA/cement ratios were applied. Plus, ACE can be operated longer with such mixtures because the open time becomes longer as the EVA/cement ratio increases. In summary, the results clearly demonstrated that EVA-modified cementitious mixtures were feasible for use as ACE materials.

## 1. Introduction

Emerging technologies are regularly being introduced to improve construction productivity and change existing construction practices. Among these evolving technologies is additive construction by extrusion (ACE). Studies related to this technology are being actively carried out, investigating the feasibility of its application in construction projects. ACE is an innovative technique that not only allows concrete structures to be built without a formwork, but also makes possible the construction of structures designed with complicated shapes [1]. The ACE technology was first applied to construction projects by Pegna in 1997 [2]. The process gained prominence in the construction field after contour crafting was introduced by Khoshnevis in 2003 [3]. Since then, ACE has been actively studied and is now being applied to small residences [4,5,6] and pedestrian bridges [7].

ACE technology can be broken down into the following independent components: 3D printers, modeling software, and printing materials (i.e., mortar). The major study areas associated with ACE can be classified into the machine, 3D modeling, and material areas [8]. Among these three research fields, few studies have been conducted on printing materials, especially as compared to the mechanical and modeling aspects. Existing studies have produced ACE materials after adding other substances based on Portland cement, fly ash, and silica fume [9,10]. There have also been cases where nonsolid ceramics such as gypsum or clay [11] were used, as well as lunar soils [12]. In a study conducted by the University of Southern California, mortar with artificial aggregates such as glass beads were employed in ACE materials [13].

Although basic research in this area has been conducted, there are currently no accepted specifications and test methods related to ACE materials. Thus, in 2018, the American Concrete Institute (ACI) organized a new committee (i.e., Committee 564: 3-D Printing with Cementitious Materials) to focus on cement materials for 3D printing. The first meeting was held on 25 March 2019 in Quebec, Canada [14]. However, more work is needed to set up appropriate specifications and test methods for ACE materials. Therefore, this research used existing studies to examine the tests required for ACE materials. Based on a literature review, it was identified that the properties of fresh ACE materials should be evaluated through a number of test methods. Specifically, from the existing research, it was identified that flowability (i.e., pumpability), extrudability (i.e., printability), buildability, and open time have frequently been tested [15,16,17].

## 2. Research Objectives

Since the importance of ACE printing materials is clear, this study, which examined cementitious mixtures for ACE processes, is both appropriate and timely. In past research on this topic, only Portland cement and various admixtures were used to produce the ACE materials. Currently, there is no research on the use of ethylene-vinyl acetate (EVA) polymers, which have widely been used to improve the performance of cementitious materials [18,19,20,21,22].

There are many kinds of polymer admixtures currently being applied in the field of construction. Among these, polymer-modified cementitious mixtures using EVA, which is a typical redispersible polymer powder, offer superior bending, excellent tensile and adhesion strength, and high resistance to the diffusion of chloride ions, oxygen, and carbon dioxide in conventional cementitious mixtures [18]. For these reasons, it is frequently employed in the construction industry. However, to date, no study has examined the potential value of EVA-modified cementitious mixtures for use as an ACE material. Thus, the present work experimentally investigated the properties of fresh EVA-modified cementitious mixtures in order to evaluate their feasibility as an ACE material.

## 3. Experiment

### 3.1. Printing Setup and Procedure forAdditive Constriction by Extrusion

A gantry-type custom-made extrusion-based 3D printer was used for the ACE test. The pumping system was a custom-made extrusion-based 3D printer consisting of a peristaltic (i.e., squeeze) pump and nozzle head. This printing material pumping system could be connected not only to the gantry, but also to a robot arm. The specifications of the peristaltic pump used in this study can be found in Table 1.

**Figure 1:**
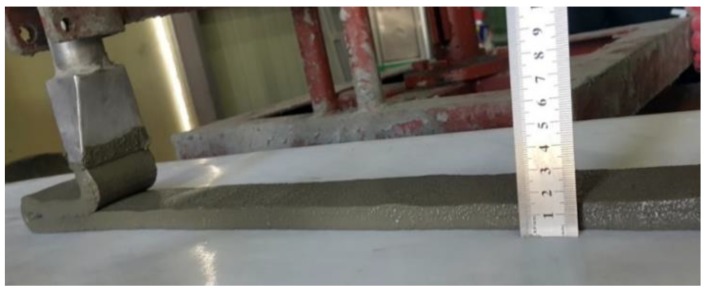
View of additive construction by extrusion test.

**Figure 2:**
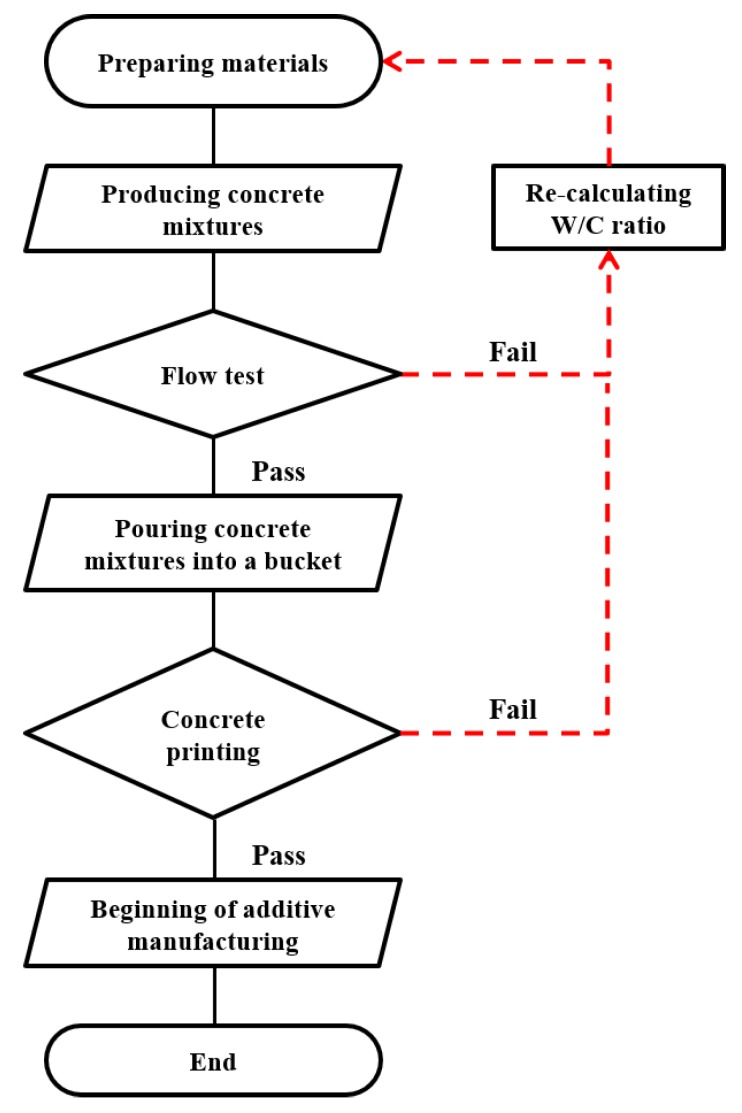
Additive construction by extrusion test procedure used in this study.

During the printing test, the lamination length for one layer was 50 cm, and the cross-section of the nozzle was 5.7 cm wide and 1.4 cm long. An image of the ACE test appears in Figure 1, and the test sequence for this study is shown in Figure 2.

### 3.2. Materials and Mix Design

#### 3.2.1. Materials

The materials used in this study included: ordinary Portland cement, EVA powder, silica sand, fly ash, silica fume, superplasticizer, and a viscosity-modifying agent. The properties of the materials used were as follows (see Table 2, Table 3, Table 4, Table 5, Table 6, Table 7 and Table 8):

#### 3.2.2. Mixture Design

**Figure 3:**
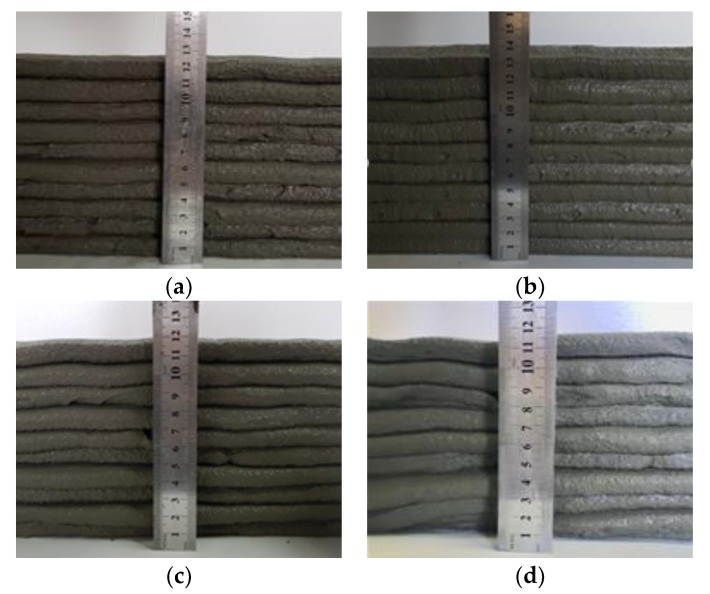
Test results for determining the flow with: (**a**) Flow of 60%; (**b**) Flow of 65%; (**c**) Flow of 70% and (**d**) Flow of 75%

Determining the optimal flow of the EVA-modified cementitious mixtures was of the utmost importance when determining the optimum mixing ratio. Thus, this study used a trial-and-error procedure to investigate the ideal flow, which met the requirements of buildability. The flow of each EVA-modified cementitious mixture was tested at 5% intervals, ranging from 55% to 75%, in order to determine the optimum state. The results of these flow tests showed that at a 55% flow, the mixture was too sticky to be dispensed from the nozzle of the 3D printer. In contrast, the mixture was easily distributed from the nozzle at a 75% flow. However, the resulting printed layers were uneven because the mixture was watery. The criteria of the optimal flow were the stacked height and the homogeneity of the layer thickness, which were optimal at a 65% flow, as shown in Figure 3.

**Figure 4:**
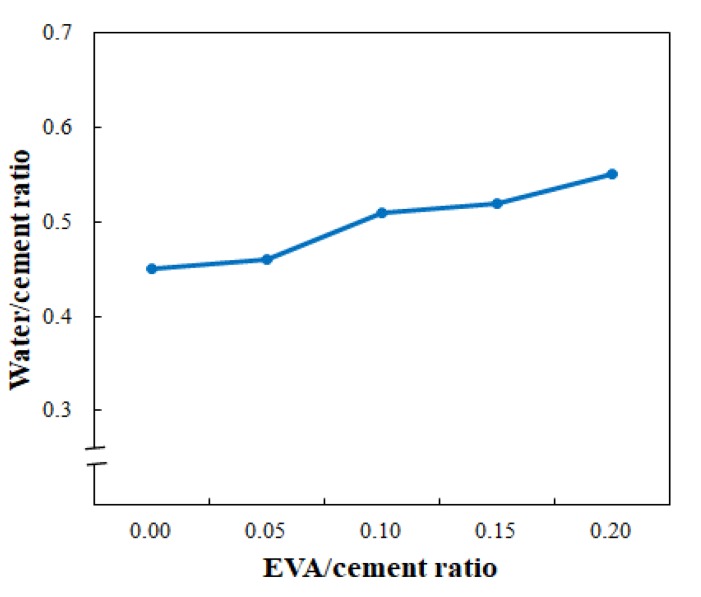
Water/cement ratio versus EVA/cement ratio.

When the EVA/cement ratio was changed, the water/cement (W/C) ratio that consistently resulted in a 65% flow was as shown in Figure 4. It was found that as the EVA/cement ratio increased, the W/C ratio also increased because EVA is a redispersible powder. The optimal mixing ratio for the EVA-modified cementitious mixture derived from a 65% flow, and the resulting W/C ratio (see Figure 4) is shown in Table 9. In Table 9, the total sum of the cement, silica sand, fly ash, and silica fume was 100 wt.%. The superplasticizer and viscosity-modifying agent are expressed in parts per hundred parts of cement (phc) because they were added in trace amounts.

## 4. Testing and Results

### 4.1. Flowability

Flowability is an indicator of the ease with which EVA-modified cementitious mixtures reach the nozzle from the tank of a 3D printer before the material is injected. It is also referred to as pumpability. There are several flowability test methods, such as vebe time, compacting factor, slump, and flow. However, if the material has a very high level of workability, the flow test method is preferable [23]. Hence, ASTM C1437-15: Standard Test Method for Flow of Hydraulic Cement Mortar was selected to evaluate the flow of the EVA-modified cementitious mixtures analyzed in the present research [24].

**Figure 5:**
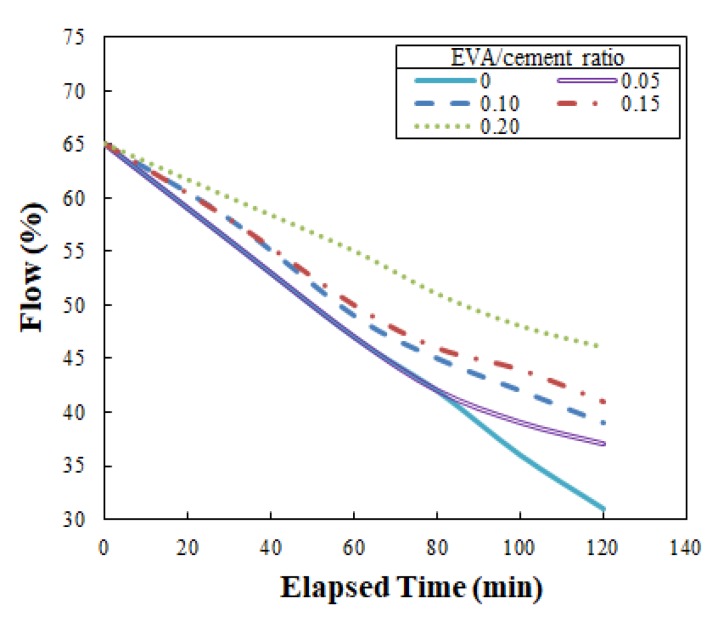
Change of the flow with elapsed time.

The optimal flow of the EVA-modified cementitious mixtures achieved using this method was 65%, showing that the flow of the mixtures produced for ACE was relatively low compared to the 110 ± 5% standard flow specified by ASTM C109/C109M-02: Testing Method for Compressive Strength of Hydraulic Cement Mortar [25]. Based on this optimal flow of 65%, the flow was measured at intervals of 30 min for the first hour, followed by intervals of 20 min for 120 min. The experiment employed in this study was based on a trial-and-error procedure using various EVA/cement ratios. Among the various ratios (ranging from 0 to 0.2), EVA—cement mixtures were not extruded from the nozzle of the 3D printer after an elapsed time of 87 mins when the EVA/cement ratio reached 0.2. Thus, flow was only tested up to 120 min. The flow test results are shown in Figure 5, indicating that the flow increased with increases in the EVA/cement ratio when the elapsed time remained the same. In other words, the loss of flow decreased when the EVA/cement ratio increased. This is a very favorable result in terms of securing flowability. The results also showed that flow consistency was improved because there was a dispersing effect of the surfactants in the polymers, which was due to the ball-bearing action of polymer particles and entrained air when the redispersible EVA powder was dispersed in water during preparation of the mixtures [18].

### 4.2. Extrudability

**Figure 6:**
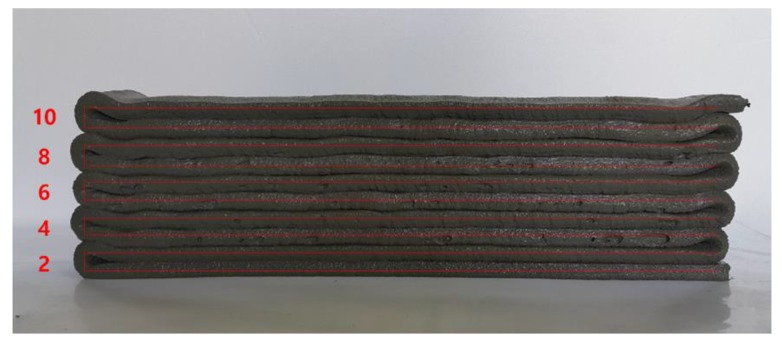
Extrudability test result with the number of printed layers.

Extrudability, also referred to as printability, is an indicator of the extent to which a printing material is smoothly and continuously extruded from the nozzle of a 3D printer. Extrudability depends on flowability and is influenced by constituents in the cementitious mixtures, including type, properties, quantity, moisture content, the presence of additives, delivery system of the printing material, and time. Extrudability is evaluated by the rate (cm/min or cm/s) at which cementitious mixtures are extruded through the nozzle head of the 3D printer. Extrudability is determined by taking the continuous length of cementitious mixture extruded and dividing by the time taken for extrusion. An image of the extrudability test is shown in Figure 6.

**Figure 7:**
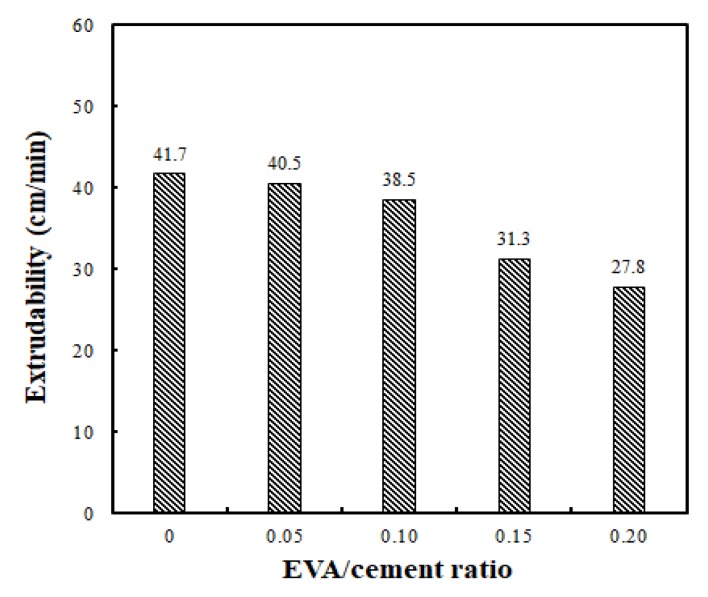
Comparison of extrudability using different EVA/cement ratios.

The printing conditions according to the EVA/cement ratio were 50 cm in horizontal length and 10 consecutive layers in height, for a total length of 500 cm. In this test, the extrusion time was measured while the feed rate of the peristaltic pump was kept constant at 1.2 rpm. The results are shown in Figure 7. At EVA/cement ratios of 0, 0.05, 0.10, 0.15, and 0.20, the extrudability levels were 41.7 cm/min, 40.5 cm/min, 38.5 cm/min, 31.3 cm/min, and 27.8 cm/min, respectively. These test results are one of many ways of examining the differences in extrudability according to the EVA/cement ratio. Naturally, increasing the feed rate of the pump would result in a faster output.

The results of this test show that the extrudability decreased when the EVA/cement ratio increased. This was due to the redispersible nature of the EVA powder; when the EVA powder was redispersed, the viscosity of the cementitious mixtures increased. When the flow was maintained at 65% while applying the EVA-modified cementitious mixtures in situ, its quality remained the same. Therefore, there was no problem with ACE because the feed rate of the 3D printer pump could be adjusted to control the extrudability.

### 4.3. Buildability

Buildability is an indicator of how high the cementitious mixtures extruded from the nozzle of the 3D printer can be stacked. This refers to the ability of a particular cementitious mixture to sustain itself as the layers are placed upon one another; thus, the test method is directly related to the number of layers that can be stacked. The buildability test in the present research was carried out by measuring the number of layers of the cementitious mixture and vertical deformation of the layer height according to the elapsed time. In this study, buildability was evaluated in the same manner as extrudability, by measuring the stacked height and height reduction after stacking 10 layers with a unit length of 50 cm per layer.

**Figure 8:**
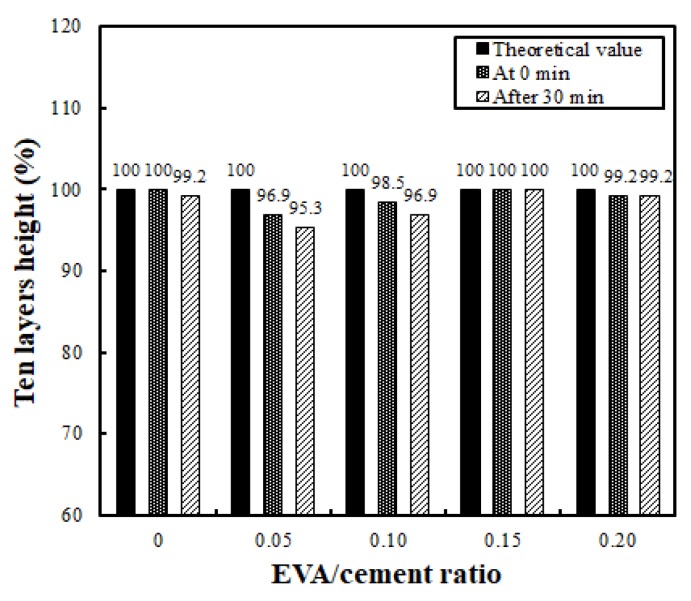
Buildability levels for different EVA/cement ratios and elapsed time.

Buildability is the most critical factor in the fresh properties of EVA-modified cementitious mixtures used for printing. A performance test is only possible if the flowability and extrudability discussed above are adequate. The buildability test in the present research was conducted by evaluating the height of a 10-layer stack with a unit length of 50 cm, both immediately after completion and once 30 min had passed. Figure 8 compares the height reduction rate immediately after being stacked and 30 min later, based on a theoretical value obtained by multiplying the height of the first initial layer by 10 (layers). The results show that the reduction rate of the stack height did not differ significantly with the EVA/cement ratio, demonstrating a relatively good buildability. The most stable buildability occurred when the EVA/cement ratio was 0.15; there was no vertical change in the stacked layers. 

**Figure 9:**
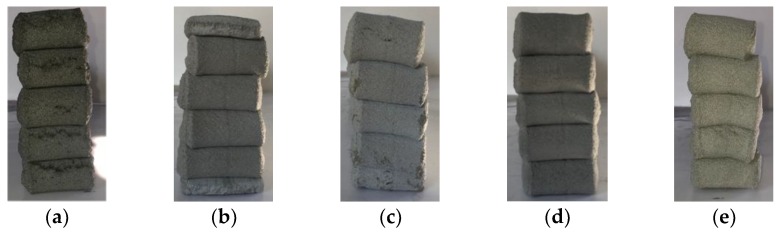
Views of buildability test at 65% flow: (**a**) EVA/cement ratio: 0; (**b**) EVA/cement ratio: 0.05; (**c**) EVA/cement ratio: 0.10; (**d**) EVA/cement ratio: 0.15 and (**e**) EVA/cement ratio: 0.20.

The results of the buildability test when the flow of the EVA-modified cementitious mixtures was 65% are shown in Figure 9. From these results, it can be seen that there were no cracks on the surface of the layer at the point where the direction of the nozzle head was changed, with the exception of when the EVA/cement ratio was 0. The best buildability occurred when the EVA/cement ratio was 0.15 because this showed the smoothest surfaces of the layers. These results are similar to reports stating that the occurrence of cohesion due to viscosity provides excellent resistance to bleeding and segregation, even though polymer-modified cementitious mixtures—as compared to ordinary cementitious mixtures—have larger flowability characteristics [18].

### 4.4. Open Time

Open time is the minimum amount of time that a material’s performance can be kept consistent. In ACE, it refers to the time beginning with extrusion and ending at the point at which extrusion becomes impossible due to decreased flowability. Open time is the best way of expressing a mixture’s changes in workability over time. It is calculated by the change in flowability over time and the flow test method employed.

**Figure 10:**
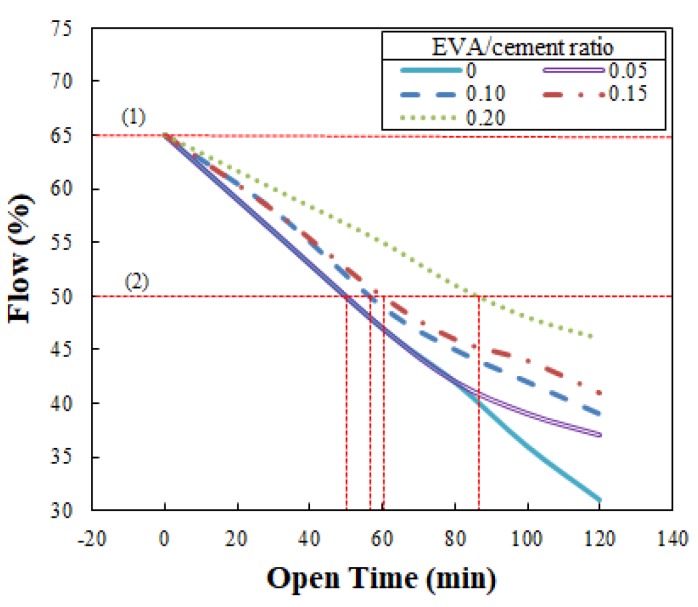
Determination of open time with the change of the flow: (1) reference point at 65% flow and (2) reference point at 50% flow.

As mentioned in Section 3.2.2, at a flow of 50%, EVA-modified cementitious mixtures cannot be extruded through the nozzles of the 3D printer. Hence, this value was used as a reference for setting the open time of the ACE. Open time can be identified by plotting a horizontal line at 50% flow, as shown in Figure 10. In Figure 10, (1) is the reference point at 65% flow and (2) is the reference point at 50% flow, indicating that extrusion is impossible. The open times obtained from the Figure 10 were 50 min, 56 min, 61 min, and 81 min at EVA/cement ratios of 0, 0.05, 0.10, 0.15, and 0.20, respectively.

As the results indicate, the open time became longer when the EVA/cement ratio increased. These results demonstrate that this time was sufficient to operate the ACE. This is similar to what was reported by Weng et al. [26], in that the initial and final settings of the mixtures were delayed when the polymer powder was increased. This phenomenon was caused by a delay in the initial setting; the initial hydration reaction of the cement was inhibited by the formation of a polymer film. These results are consistent with a previous study stating that the setting of polymer-modified cementitious mixtures can to some extent be delayed as compared to conventional cementitious mixtures, and is dependent on the polymer type and polymer/cement ratio [18].

## 5. Conclusions

In this study, the fresh properties of EVA-modified cementitious mixtures were experimentally investigated to determine their feasibility as ACE materials. The results obtained can be summarized as follows:

(1) Extrudability and buildability tests were conducted by a trial-and-error procedure to determine the optimal ratios; the optimized flow was 65% for both performance indicators.
(2) The W/C ratio increased when the EVA/cement ratio was increased to obtain an optimal flow of 65%, which was due to the redispersible nature of EVA.
(3) At the same elapsed time, the flow increased when the EVA/cement ratio increased, which was particularly beneficial in terms of securing flowability.
(4) Extrudability is somewhat reduced as the EVA/cement ratio increased. However, if the flow of the EVA–cement mixture is maintained at 65% when applied onsite, printing can be conducted without any problems by controlling the feed rate of the 3D printer pump.
(5) There was no significant difference in stack height reduction rate, although the EVA/cement ratio was different; thus, the buildability was found to be good. In addition, buildability was superlative when the EVA/cement ratio was 0.15 because there was no decrease in stack height and the smoothest surface layers were obtained.
(6) The open time became longer when the EVA/cement ratio increased. These results show that the formation of a polymer film, which inhibits the initial hydration reaction of the cement, is advantageous for securing the ACE operation time.

According to the results of the property tests, EVA-modified cementitious mixtures are feasible for use as ACE materials.

## Figures and Tables

**Table 1:** Peristaltic pump specifications.

Inner Diameter	Flow	Power	RPM
32 mm	3.4 ton/h (max)	2HP-single (220 V)	30–60

**Table 2:** Properties of ordinary Portland cement (Type I).

Density (g/cm^3^)	pH (Wet Cement)	Vapor Pressure (mmHg at 20 °C)	Chemical Composition (%)			Specific Surface (cm^2^/g)
			MgO	SO_3_	Loss on Ignition	
3.14	12	0	2.34	2.97	2.76	3630

**Table 3:** Properties of EVA powder.

Solids Content (%)	Ash Content (%)	Bulk Density (kg/m^3^)	Particle Size after Redispersion (μm)	Minimum Film-Forming Temperature (°C)	Protective Colloid	Film Properties of The Redispersion
98–100	9–13	470–570	0.5–8.0	4	Polyvinyl alcohol (PVA)	Cloudy, tough-elastic

**Table 4:** Properties of silica sand.

Size (mm)	Apparent Density	Purity (%)	Water Content (%)
0.08	1.57	97.3	<0.1

**Table 5:** Properties of fly ash.

Density (g/cm^3^)	SiO_2_ (%)	Loss on Ignition (%)	Specific Surface (cm^2^/g)
2.22	56.4	3.2	3651

**Table 6:** Properties of silica fume.

SiO_2_ (%)	H_2_O (%)	Loss on Ignition (%)	Bulk Density-Undensified (kg/m^3^)	Bulk Density-Densified (kg/m^3^)	Specific Surface (cm^2^/g)
96.7	<1.0	<3.0	200–350	600–700	157,700

**Table 7:** Properties of superplasticizer.

Specific Gravity (20 °C)	pH	Alkali Content (kg/m^3^)	Chloride Content (kg/m^3^)
1.05 ± 0.05	5.0 ± 2.0	0.03	0.03 × 10^−3^

**Table 8:** Properties of viscosity modifying agent.

Appearance	pH	Concentration (%)	Bulk Density (kg/m^3^)	Viscosity (mPa·s, 25 °C)	Moisture Content (%)	Particle Size (0.074 mm) %
White powder	8.0–10.0	8.5	430	45,000	≤12	99

**Table 9:** Mixture proportions of EVA-modified cementitious mixtures.

EVA/Cement Ratio	Water/Cement Ratio	Cement (wt.%)	Silica Sand (wt.%)	Fly Ash (wt.%)	Silica Fume (wt.%)	Superplasticizer (phc *)	Viscosity-Modifying Agent (phc *)
0	0.45	28	60	8	4	(1)	(0.05)
0.05	0.46						
0.10	0.51						
0.15	0.52						
0.20	0.55						

* Parts per hundred parts of cement.
